# First record of the nematode *Libyostrongylus dentatus* Hoberg, Lloyd & Omar, 1995 (Trichostrongylidae) in ostriches (*Struthio camelus* Linnaeus, 1758) (Struthionidae) outside the Americas

**DOI:** 10.1186/s13071-018-2815-7

**Published:** 2018-04-13

**Authors:** Josiana Gomes de Andrade, Bersissa Kumsa, Dinka Ayana, Ricardo Augusto Mendonça Vieira, Clóvis de Paula Santos, Alena Mayo Iñiguez, Renato Augusto DaMatta

**Affiliations:** 1 Laboratório de Biologia Celular e Tecidual, Centro de Biociências e Biotecnologia, Universidade Estadual do Norte Fluminense Darcy Ribeiro, Av. Alberto Lamego, 2000, Parque Califórnia, Campos dos Goytacazes, RJ 28013-602 Brazil; 2 Department of Parasitology and Pathology, College of Veterinary Medicine and Agriculture, Addis Ababa University, P.O. Box 34, Bishoftu, Ethiopia; 3 Laboratório de Zootecnia, Centro de Ciências e Tecnologias Agropecuárias, Universidade Estadual do Norte Fluminense Darcy Ribeiro, Campos dos Goytacazes, RJ 28013-602 Brazil; 4 LABTRIP, Instituto Oswaldo Cruz, Fundação Oswaldo Cruz-Fiocruz, Pavilhão Rocha Lima, Sala 518, Av. Brasil 4365, Rio de Janeiro, RJ 21045-900 Brazil

**Keywords:** *Libyostrongylus* spp., *Struthio molybdophanes*, Ethiopian ostriches, Phylogeny, Internal transcribed spacer

## Abstract

**Background:**

*Libyostrongylus douglassii*, *Libyostrongylus dentatus* and *Libyostrongylus magnus* are nematodes that infect ostriches. The first species has been identified in ostriches from Africa, Europe, Americas and Oceania. Although the natural range of ostriches is Africa, *L. dentatus* was first described in birds from the USA and later identified in Brazil, where co-infections with *L. douglassii* have been commonly reported. *Libyostrongylus magnus* is known from the original description only. There are a few reports on infections with *L. douglassii* in ostriches from Africa and all farmed birds examined are from the southern region of the continent. The aim of this report was to verify *Libyostrongylus* spp. infections in wild ostriches from Ethiopia. Fecal samples from ostriches, *Struthio molybdophanes*, were collected and submitted to coproculture. Infective larvae were identified to the species level based on general morphology and morphometry. In addition, phylogenetic analysis of the first and second internal transcribed spacer (ITS1 and ITS2) of the nuclear ribosomal DNA was performed.

**Results:**

Infective larvae from Ethiopian ostriches had the morphological characteristics of *L. dentatus*. Confidence interval estimate for sheath tail length from Ethiopian *Libyostrongylus* sp. isolates overlapped one for Brazilian *L. dentatus*. Neighbor-joining and Maximum Likelihood phylogenetic trees based on sequences of the ITS1 and ITS2 regions revealed that the Ethiopian samples belong to the *L. dentatus* species clade. Monospecific infections with *L. dentatus* were confirmed in Ethiopian wild ostriches, opposed to the co-infections typically found in the Americas.

**Conclusions:**

To our knowledge, this is the first record of *L. dentatus* from African ostriches, the region from which this parasite originated.

## Background

Ostriches, *Struthio camelus* Linnaeus, 1758 (Aves: Struthionidae), are infected by several species of helminths [1, 2]. In recent years, studies on nematodes of the genus *Libyostrongylus*, commonly known as wireworms, have intensified [3–6]. The genus *Libyostrongylus* comprises *Libyostrongylus dentatus* Hoberg, Lloyd & Omar, 1995, *Libyostrongylus douglassii* Lane, 1923 [7] and *Libyostrongylus magnus* Gilbert, 1937. Species of this genus inhabit the proventriculus and ventriculus of ostriches [4, 8, 9] and feed on blood [1, 10]. Infection with *L. douglassii* causes a disease known as rotten stomach, responsible for high mortality rates in young, and occasionally, adult birds [10–12]. The pathogenic outcomes of infections with *L. dentatus* [13] and *L. magnus* are not determined.

*Libyostrongylus douglassii* has been found in ostriches raised in South Africa [7, 14, 15], Oceania [1, 16], the Americas [4–6, 9, 13, 17–20] and Europe [2, 21–26]. Although Africa is the continent of origin of ostriches, *L. dentatus* has never been reported in this continent. This species was first described in birds raised in the USA [13] and later reported in Brazil [4–6, 9, 17, 18]. Due to morphological similarities of *L. douglassii* and *L. dentatus*, it was suggested that the latter has been overlooked [9, 13, 18]. *Libyostrongylus magnus* was described in the Ukraine in ostriches originating from Ethiopia [27, 28]. The females of this species are smaller than males and the ovejector is larger than that in *L. douglassii* and *L. dentatus*. No morphological and genetic characterizations of *L. magnus* infective larvae are available [29] and there are no recent reports of this species.

Although *L. douglassii* was described from material in South Africa [7], as far as we know, there are only two other published reports of this nematode from ostriches born and maintained in farmed ostriches from Africa. One is the first article on the resistance of this nematode to levamisole [14], and the other reports the parasite in ostriches from the Highveld region of Zimbabwe [15]. There are no reports of *Libyostrongylus* spp. infections in wild ostriches from South Africa, or from other African countries. This paper reports, for the first time, infections with *Libyostrongylus* spp. in wild Ethiopian ostriches.

## Methods

### Samples

Faeces from 5 ostriches (*Struthio molybdophanes* Reichenow, 1883) were collected at Abijata-Shalla Lakes National Park located in Oromia Regional State, Ethiopia (geographical location: 7°50'N, 38°50'E). Faeces were cultured [30] to obtain infective larvae (L3), at the Laboratory of Veterinary Parasitology, College of Veterinary Medicine and Agriculture, Addis Ababa University, Bishoftu. L3 were preserved in 70% ethanol and shipped to Brazil.

### Morphology of infective larvae

Four hundred L3 from each of the 5 hosts were identified under a microscope based on the general morphology of L3, especially the features of the tail, which differentiate *L. douglassii* from *L. dentatus* [18]. From this group containing 400 L3, the total length and the sheath tail length was measured for 100 L3 [18]. Images of L3 were captured using a Zeiss Axioplan microscope (Jena, Germany) equipped with differential interference contrast, an AxioCam Mrc5 and the Axiovision program (Germany) at the Universidade Estadual do Norte Fluminense Darcy Ribeiro.

### Statistical analysis

Ethiopian *Libyostrongylus* sp. L3 were investigated in detail by employing statistical analyses using confidence intervals (CI) of measurements of total length and sheath tail length of at least 100 specimens of *L. douglassii* and *L. dentatus* from 14 locations in Brazil [5] and of the 100 specimens from Ethiopia that were measured. The L3 larvae of *L. douglassii* and *L. dentatus* from Brazil were fixed in 70% ethanol; these were collected in previous studies performed on Brazilian farmed ostriches [5, 6]. The CI of the measurements of L3 were statistically estimated using the following mixed model:

$$ {Y}_{ij k}=\mu +{\alpha}_i+{\beta}_j+{\alpha \beta}_{ij}+{e}_{ij k} $$

where *Y*_*ijk*_ is the observation related to the *k*-th parasite from *j*-th region of the *i*-th species. The fixed effects are described in Greek letters including the intercept of the model (*μ* ). The error term is represented by *e*_*ijk*_ assumed normal, independent and identically distributed with mean zero and variance σ^2^. The variance-covariance structure was tested for normality and independence for the random errors according to published recommendations [31]. The model was fitted to data by means of the PROC MIXED procedure of SAS (v.9, SAS System Inc., Cary, NC, USA) [31]. The variance-covariance structure that most likely described the data was the diagonal heterogeneous variances. Point and interval estimates for total L3 size and sheath tail length for *L. douglassii* and *L. dentatus* from Brazil and the 500 specimens from Ethiopia were obtained by assuming a 99% confidence level. Results are presented as least squares means and the lower and upper limits of the 99% CI (99% CI) presented in parentheses. Nonetheless, for statistical comparison purposes using the adjusted Tukey-Cramer test, we adopted the type I error rate of 0.02 to avoid spurious positive statistical findings [32].

### DNA extraction, PCR amplification and sequencing

A pool of 50 L3 specimens from each of the 5 hosts, in duplicates, was used for DNA extraction in three steps: samples were frozen in liquid nitrogen, submitted to digestion with proteinase K [33] and then a QIamp® DNA mini kit (Qiagen, Hilden, Germany) was used according to the manufacturer's instructions. Samples were observed under an optical microscope to assure the digestion of the cuticles of the larvae. The DNA was quantified in a ND-1000 NanoDrop Spectrophotometer (Thermo Fisher Scientific, Wilmington, USA). The first and the second internal transcribed spacer regions (ITS1 and ITS2) were amplified by PCR using the primers NC5 and NC13 and NC2 and NC13, respectively, as described elsewhere [34, 35]. The PCR reaction volume was 25 μl containing 10 mM Tris-HCl (pH 8.0), 50 mM KCl, 3 mM of MgCl_2,_ 0.2 mM of each dNTP, 20 ρmol of each primer, 1 unit of Platinum® *Taq* DNA polymerase (Invitrogen, Carlsbad, USA) and 5 μl of extracted DNA. The reactions were subjected to an initial cycle of 5 min at 94 °C, followed by 40 cycles of 94 °C for 40 s, 55 °C for 40 s, 72 °C for 40 s, and 72 °C for 7 min, in a thermal controller Mastercycler ep system (Eppendorf, Hamburg, Germany). PCR products were electrophoresed on 2.0% agarose gels and visualized using GelRed nucleic acid gel stain (Biotium, Hayward, USA). For PCR product purification, a MinElute PCR purification kit (Qiagen) was used according to the manufacturer’s protocol. Amplicons were directly sequenced in both directions using Big Dye Terminator v3.1 Cycle Sequencing Ready reaction kit (Applied Biosystems, Foster City, USA) as recommended by the supplier in a 3100 Automated DNA Sequencer (Applied Biosystems) located on the RPT01A/IOC- Fiocruz sequencing platform.

### Sequence analysis

DNA sequences were analyzed, edited and aligned using Pairwise/Blast/NCBI, DNASTAR Lasergene SeqMan v7.0.0, Bio Edit v7.2.5 [36] and ClustalX version 2.0 [37]. ITS1 and ITS2 sequences were submitted to the GenBank database under the accession numbers KY991371- KY991373. The genetic distances and phylogenetic trees were estimated using MEGA version 7 [38]. Genetic distances were calculated using the Kimura 2-parameter (K2P) model [39], complete deletion treatment and standard error estimated (SE) by a bootstrap of 1000 replicates. ITS1 and ITS2 phylogenetic trees were constructed using Neighbor-Joining (NJ) with K2P model [39], and Maximum Likelihood (ML) with Tamura 3-parameter model (T92) [40] plus gamma distribution (G), as selected by the best-fitting model of DNA substitution using the Bayesian information criterion. Complete deletion parameters and 1000 bootstrap replicates were applied.

All ITS1 and ITS2 *Libyostrongylus* spp. sequences available in GenBank were used in the dataset for phylogenetic analyses. In addition, three sequences of each species of *Trichostrongylus* spp., *Haemonchus* spp., *Teladorsagia* sp., *Ostertagia* sp., *Spiculopteragia* sp. and *Cooperia* spp. (all belonging to the superfamily Trichostrongyloidea) were included in the dataset. *Nematodirus battus* (Molineoidae) was designated as the outgroup.

## Results

**Fig. 1:**
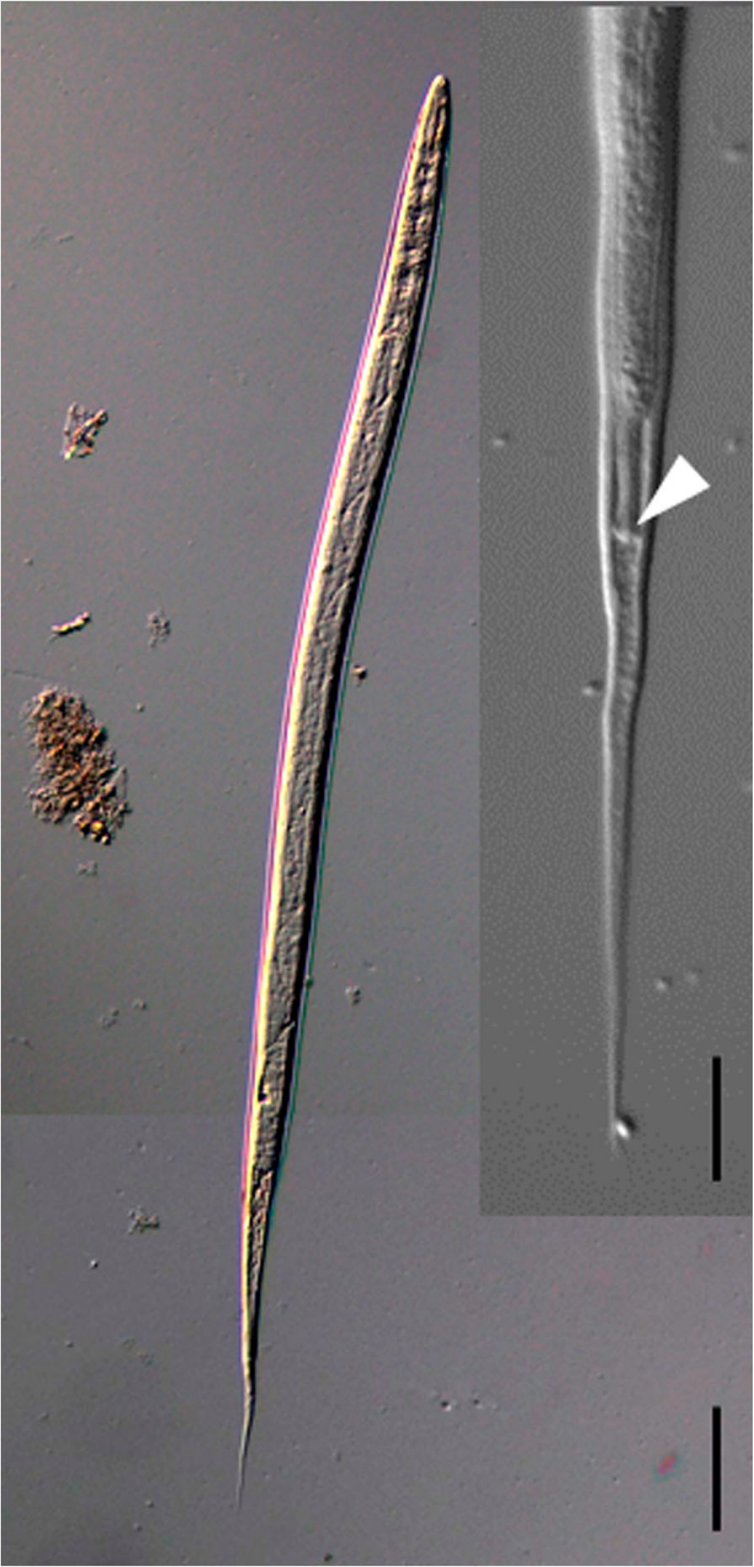
Photomicrographs of infective L3 larvae of *Libyostrongylus* sp. sampled in Ethiopia under differential interference contrast microscopy. Total view and tail of a larva with the characteristic knob (inset, arrowhead). *Scale-bar*: 40 μm (inset, 10 μm)

Morphological examination of the L3 samples from Ethiopian ostriches revealed a knob at the tail of larvae and a long and filamentous sheath tail (Fig. 1). L3 with short sheath tail and acute termination were not found in these samples. The statistical analysis of morphometric measurements of L3 revealed that the interaction effect between species and location (*αβ*_*ij*_) was significant (F-test, residual *df* = 347, *P* = 0.002), and the effects were split. Only one comparison between locations based on 99% CI for total length between Brazilian larvae of *L. douglassii* and *L. dentatus* was significant, whereas comparisons regarding the sheath tail length of both Brazilian larvae from the 14 Brazilian locations were all significant (Table 1). The 99% CI of the sheath tail length of *Libyostrongylus* sp. L3 samples obtained from Ethiopia compared to *L. dentatus* from 14 Brazilian locations were similar in 8 locations and different in the 6 remaining. The total length of the L3 between Ethiopian *Libyostrongylus* sp. and *L. dentatus* from Brazil differed in only 5 locations (Table 1).

**Table 1:** Least squares 99% confidence intervals (means ± half amplitude of 99% CI) for the total length and sheath tail length (in μm) of infective larvae of *L. douglassii* and *L. dentatus* at different Brazilian regions (origin of samples as described [4, 6]) and *Libyostrongylus* sp. from Ethiopia

Origin	Total length					Sheath tail length				
	*L. douglassii*	*L. dentatus*	*df*, *P*-value^a^	*L.* sp.	*df*, *P*-value^b^	*L. douglassii*	*L. dentatus*	*df*, *P* -value^a^	*L.* sp.	*df*, *P*-value^b^
Paraná 1	786 ± 17	813 ± 36	98, 0.993	809 ± 5	101, 1.000	32 ± 2	76 ± 6	21.5, <0.001	64 ± 1	18.9, 0.002
Paraná 2	743 ± 48	755 ± 65	15, 1.000		15.2, 0.770	35 ± 10	74 ± 20	8.9, <0.001		5.1, 0.892
Mato Grosso	816 ± 16	833 ± 31	96, 1.000		75.2, 0.957	33 ± 2	76 ± 7	25.3, <0.001		90.1, 0.020
São Paulo 1	941 ± 44	911 ± 21	68, 0.998		101, <0.001	29 ± 4	75 ± 2	25.8, <0.001		22.5, <0.001
São Paulo 2	800 ± 21	760 ± 40	98, 0.845		101, 0.260	32 ± 2	69 ± 4	27.3, <0.001		24.7, 0.345
São Paulo 3	867 ± 12	839 ± 37	94, 0.981		97.1, 0.939	33 ± 1	71 ± 7	9.2, <0.001		8.8, 0.393
Santa Catarina	773 ± 17	737 ± 66	98, 1.000		99, 0.478	37 ± 2	81 ± 6	9.1, <0.001		5.9, 0.003
Espírito Santo	811 ± 15	830 ± 27	98, 0.999		104, 0.969	32 ± 2	72 ± 5	32.2, <0.001		27.7, 0.036
Minas Gerais 1	845 ± 20	868 ± 23	98, 0.983		106, <0.001	31 ± 2	73 ± 4	59.6, <0.001		54.1, <0.001
Minas Gerais 2	840 ± 26	854 ± 27	97, 1.000		103, 0.011	28 ± 2	67 ± 4	66.8, <0.001		55.6, 0.997
Minas Gerais 3	766 ± 25	861 ± 29	98, <0.001		103, 0.003	29 ± 2	70 ± 4	58.7, <0.001		51.6, 0.059
Rio de Janeiro 1	843 ± 20	883 ± 113	98, 1.000		98.3, 0.995	34 ± 2	82 ± 30	23.6, <0.001		2.1, 0.212
Rio de Janeiro 2	767 ± 28	829 ± 35	98, 0.083		102, 0.999	35 ± 3	73 ± 6	57.2, <0.001		41.9, 0.016
Rio de Janeiro 3	691 ± 19	732 ± 26	98, 0.203		102, <0.001	28 ± 1	65 ± 3	47.6, <0.001		44.0, 1.000

^a^*P-*values for contrasts (adjusted Tukey-Cramer) between Brazilian species split within regions (within the same row) for both total length and sheath tail length, e.g. *L. douglassii vs L. dentatus* within Paraná 1 region, and so on. *P* < 0.02 indicate statistical significance

^b^*P*-values for contrasts (adjusted Tukey-Cramer) between Brazilian *L. dentatus* and *Libyostrongylus* sp. from Ethiopia within the respective region for both total length and sheath tail length, e.g. Brazilian *L. dentatus vs* Ethiopian *Libyostrongylus* sp. within Paraná 1 region, and so on

*Abbreviations*: *df* degrees of freedom, *L.* sp. Ethiopian *Libyostrongylus* sp.

Although the efficiency of DNA extraction was low, possible due to the poor preservation of the Ethiopian L3 in 70% ethanol, the DNA was successfully extracted from larvae pool of two ostrich samples (2.1 and 4.1). Two partial ITS1 sequences from samples 2.1 and 4.1 (451 and 490 bp, respectively); and one complete ITS2 sequence (237 bp) from 2.1 sample were obtained. The ITS1 alignment of 451 bp showed that the Ethiopian *Libyostrongylus* sp. sequences were identical. Ethiopian *Libyostrongylus* sp. sequences also had an insertion of 26 bp when compared to Brazilian *L. douglassii* ITS1 sequence, as demonstrated previously [3].

ITS1 genetic distance between *Libyostrongylus* sp. from Ethiopia and Brazilian *L. dentatus* was 0.003 (SE = 0.002), while between *Libyostrongylus* sp. from Ethiopia and Brazilian *L. douglassii* was 0.090 (SE = 0.017), which was the same genetic distance between *L. dentatus* and *L. douglassii* from Brazil.

ITS2 genetic distance between *Libyostrongylus* sp. from Ethiopia and Brazilian *L. dentatus* was 0.002 (SE = 0.002), while between Brazilian *L. douglassii* was 0.073 (SE = 0.020), which was the same genetic distance between Brazilian *L. dentatus* and *L. douglassii*. A higher value of genetic distance (0.100; SE = 0.025) was obtained when comparing ITS2 sequences from Ethiopian samples with a *Libyostrongylus* sp. sample of gorilla faeces from Cameroon (JX159958) [41], which coincides with the value obtained between the gorilla *Libyostrongylus* sp. and *L. dentatus* from Brazil. In addition, higher values of genetic distance were obtained when comparing *L. dentatus* and *L. douglassii* from Brazil (0.073; SE = 0.020).

**Fig. 2:**
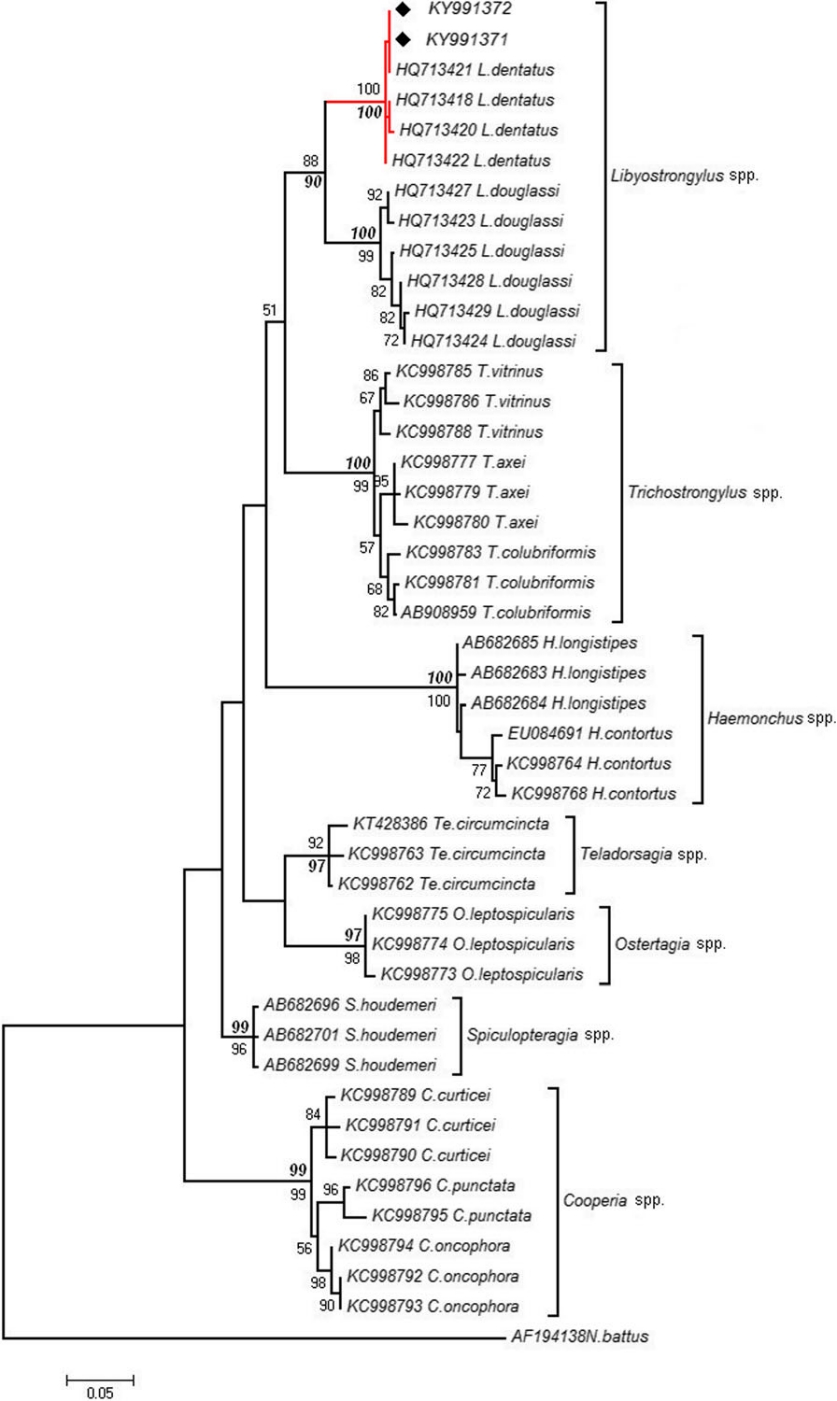
Maximum Likelihood (ML) phylogenetic tree inferred from ITS1 sequences for Ethiopian *Libyostrongylus* sp. (this study) and representative GenBank sequences for species of related families (Trichostrongylidae, Cooperiidae). Bootstrap values (> 50%) for nodal support correspond to ML analysis using T92 + G model (italic and bold font) and NJ analysis with K2P model (regular font). *Libyostrongylus dentatus* clade is indicated in red; filled diamonds correspond to the newly generated sequences for Ethiopian *Libyostrongylus* sp.

NJ and ML trees based on the ITS1 dataset showed that both *Libyostrongylus* sp. sequences from Ethiopia clustered with *L. dentatus* from Brazil in a monophyletic clade with maximum bootstrap values (NJ and ML = 100%) (Fig. 2), while *L. douglassii* clustered in another monophyletic clade with high bootstrap values (ML = 99%, NJ = 100%). All *Libyostrongylus* spp. sequences formed a well-supported clade (ML = 88%, NJ = 90%). In addition, genus-specific clades with strong support were detected for *Trichostrongylus* spp., *Haemonchus* spp., *Teladorsagia circumcincta*, *Ostertagia leptospicularis*, *Spiculopteragia houdemeri* and *Cooperia* spp. (ML = 92–100%, NJ = 97–100%) (Fig. 2).

**Fig. 3:**
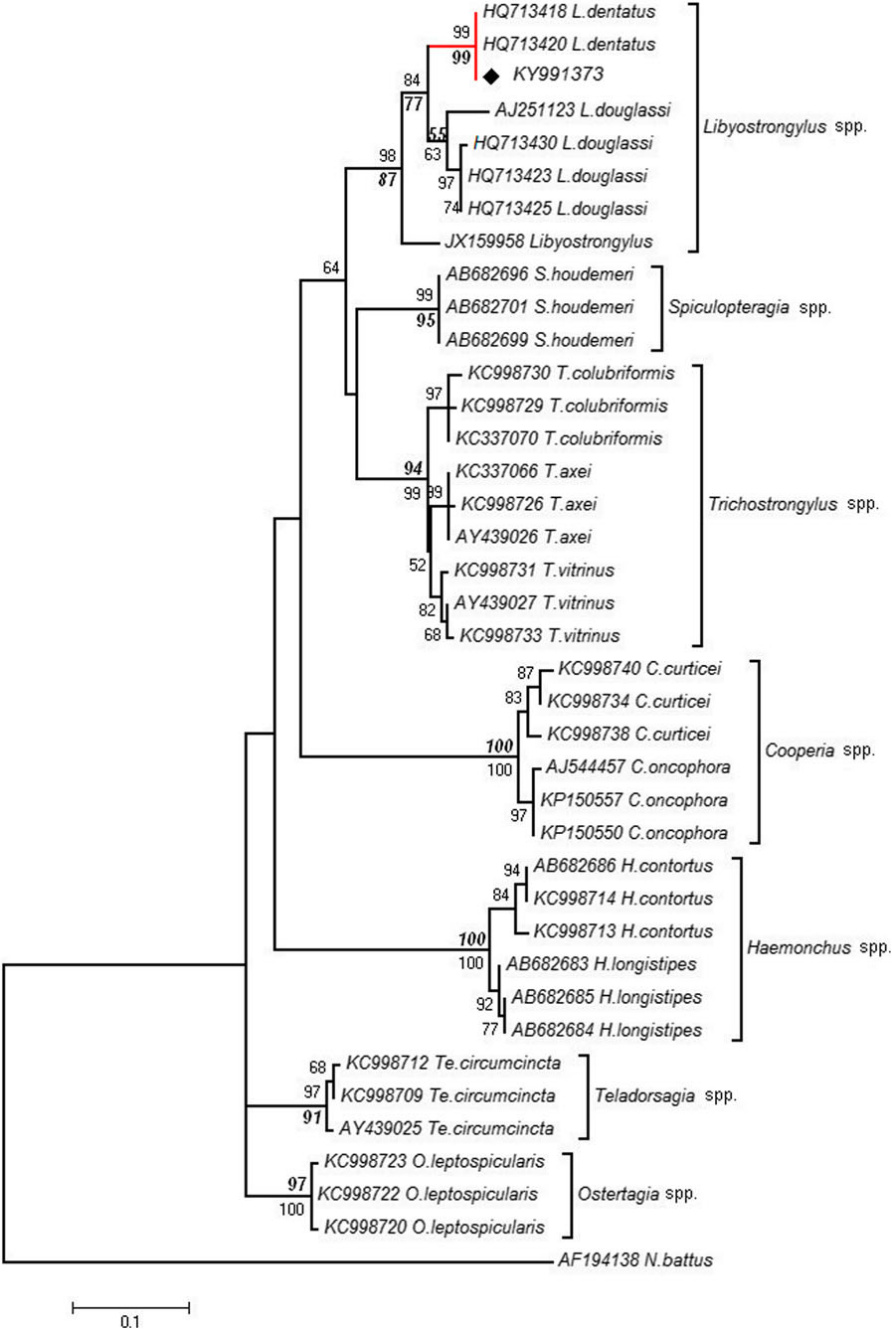
Maximum Likelihood (ML) phylogenetic tree inferred from ITS2 sequences for Ethiopian *Libyostrongylus* sp. (this study) and representative GenBank sequences for species of related families (Trichostrongylidae, Cooperiidae). Bootstrap values (> 50%) for nodal support correspond to ML analysis using T92 + G model (italic and bold font) and NJ analysis with K2P model (regular font). *Libyostrongylus dentatus* clade is indicated in red: the filled diamond corresponds to the newly generated sequence for Ethiopian *Libyostrongylus* sp.

Similarly, the ITS2 ML and NJ trees revealed that *Libyostrongylus* sp. from Ethiopia grouped with *L. dentatus* in a monophyletic clade with high bootstrap support (ML and NJ = 99%) (Fig. 3). *Libyostrongylus douglassii* sequences also grouped in a monophyletic clade but with low bootstrap support (ML = 55%, NJ = 63%), showing a strongly-supported subgroup comprising only Brazilian *L. douglassii* (ML = 97%). The *Libyostrongylus* sp. sequence from gorilla (GenBank: JX159958) was resolved as basal to *Libyostrongylus* spp. clade (Fig. 3), but still clustered in the genus-level clade, with strong and moderate support (ML = 98%, NJ = 87%). Furthermore, genus-specific clusters with strong support were detected for *Trichostrongylus* spp., *Spiculopteragia houdemeri*, *Cooperia* spp., *Haemonchus* spp., *Teladorsagia circumcincta* and Ostertagia *leptospicularis* (ML = 97–100%, NJ = 81–100%).

## Discussion

*Libyostrongylus* spp. infection in ostriches is widely distributed in the world, being a major problem for commercial production of these animals. There is relative lack of data concerning this infection in African countries and none in wild ostriches. In this study we examined *Libyostrongylus* spp. parasitism in wild ostriches from Ethiopia. Morphological and morphometric data for L3 larvae demonstrated the presence of only one species of *Libyostrongylus*. i.e. *L. dentatus*, in these ostriches that was supported by genetic characterization.

The total length of the L3 is a characteristic that helps the differentiation of *L. douglassii* from *L. dentatus* but presents an elevated standard deviation that may lead to error [18]. This was further demonstrated in samples from different Brazilian locations [5] in a two-year systematic sampling [6], clearly indicating that sheath tail length is the best morphological parameter to distinguish these species. The statistical analysis presented herein also confirmed that sheath tail length is the best morphological character to discriminate between *L. douglassii* and *L. dentatus*.

The observation of the knob at the posterior end of the L3 from Ethiopia clearly indicates that this material belongs to the genus *Libyostrongylus* [1, 18]. The presence of the long filamentous sheath tail found in Ethiopian samples indicates that the species observed was *L. dentatus*. This was confirmed by statistical analysis of the sheath tail length that showed a greater similarity between Ethiopian *Libyostrongylus* sp. and Brazilian *L. dentatus* than Brazilian *L. douglassii*, further suggesting that the Ethiopian samples belong to *L. dentatus*.

Molecular data were used to confirm morphological data. *Libyostrongylus* sp. sequences from the Ethiopian hosts clustered with *L. dentatus* sequences from Brazil. Ethiopian samples presented a 26 bp insertion in the ITS1 region that is present only in *L. dentatus* [3]. Phylogenetic trees based on ITS1 and ITS2 datasets showed a monophyletic group comprising *Libyostrongylus* sp. from Ethiopia and *L. dentatus*, with high bootstrap support. These robust phylogenetic results, together with the morphological data, confirm that *Libyostrongylus* sp. from Ethiopia was *L. dentatus* and not another known or new *Libyostrongylus* species.

An ITS2 *Libyostrongylus* sp. sequence of gorilla faeces from Cameroon [41] clustered with strong support with *Libyostrongylus* spp. in the present study. Hamad et al. [41] suggested that the detection of *Libyostrongylus* sp. in gorilla fecal samples was probably due to environmental contamination or consumption of food contaminated by larvae from an ostrich. Although the ITS2 sequence from gorilla faeces belonged to the genus *Libyostrongylus* in our analysis, high genetic distances relative to both *L. douglassii* and *L. dentatus* were found, not allowing the identification of the species. An explanation is that the gorilla fecal sample sequence could represent a species of the genus *Paralibyostrongylus*, which is closely related to the genus *Libyostrongylus*. *Paralibyostrongylus* was regarded as a synonym of *Libyostrongylus* by Chabaud [42]. However, other authors have considered *Libyostrongylus* and *Paralibyostrongylus* as distinct genera that belong to the same subfamily, Libyostrongilinae [43]*.* Gorillas are the hosts of *Paralibyostrongylus hebrenicutus* and *P. kalinae* [44–46]; no molecular data for these species are available. Another possibility is that the sequence (JX159958) is another *Libyostrongylus* species not yet morphologically characterized. Further molecular analysis of *Paralibyostrongylus* spp. is necessary to verify if the gorilla fecal sample sequence belongs to the genus *Paralibyostrongylus* or represents another *Libyostrongylus* species.

*Libyostrongylus magnus* was described in ostriches from Ethiopia [13, 27, 28]. However, the morphological and genetic characterization presented in our study identified *L. dentatus* from the Ethiopian samples. The absence of *L. magnus* in these samples may be explained by the host subspecies studied. We identified *L. dentatus* in *S. molybdophanes*, while *L. magnus* was described in *S. c. camelus* [13]. The distribution of the two species may possibly vary according to the ostrich subspecies as proposed previously [13].

To our knowledge, this is the first record of *L. dentatus* outside the Americas, and as a monospecific infection. In our previous studies on farmed ostriches in Brazil, monospecific *Libyostrongylus* infection was never detected [3, 6]. A population dynamics study of *L. dentatus* and *L. douglassii* was performed for two years, analyzing about 192,000 L3 from 40 ostriches, and a monospecific *Libyostrongylus* sp. infection was never recorded in those birds. The co-infection condition (*L. dentatus* + *L. douglassii*) was always found, with a general predominance of *L. douglassii* [6]. In addition, ostriches from 13 Brazilian farms from distinct regions of the country were also examined and, again, a monospecific infection by *Libyostrogylus* sp. was never recorded [3]. Furthermore, in both studies, the species that dominates the co-infection was *L. douglassii*, not *L. dentatus*. In the present study, only *L. dentatus* was found in the 2000 L3 examined, indicating that this is the first record of a monospecific *Libyostrongylus* sp. infection ever reported. More ostriches from Ethiopia, and from distinct African regions, need to be sampled to understand the infection patterns of *Libyostrongylus* spp.

## Conclusions

To our knowledge, this is the first record of *L. dentatus* infecting ostriches outside the Americas. It is also the first report of *L. dentatus* infection in wild ostriches from an Ethiopian region. The study demonstrates a scenario of monospecific infections with *L. dentatus* in Ethiopian wild ostriches, contrary to the typical observation of co-infections in farmed ostriches from the Americas.

## Abbreviations

CI: Confidence intervals
G: Gamma distribution
ITS1: First internal transcribed spacer
ITS2: Second internal transcribed spacer
L3: Infective third-stage larvae
ML: Maximum likelihood
NJ: Neighbor-joining
SE: Standard error
